# Effectiveness of cleaning protocols for temporary methacrylate-based resin cements in root canal dentin

**DOI:** 10.1590/1807-3107bor-2026.vol40.081

**Published:** 2026-10-23

**Authors:** Vinicius Leite Rosa-E-Silva, Fábio Augusto Soares da Silva, Guilherme Nilson Alves-Dos-Santos, Helena Cristina de Assis, Aline Evangelista Souza-Gabriel, Yara Teresinha Corrêa Silva-Sousa, Manoel Damião Sousa-Neto, Fabiane Carneiro Lopes-Olhê, Jardel Francisco Mazzi-Chaves

**Affiliations:** (a) Universidade de São Paulo – USP, School of Dentistry of Ribeirão Preto, Department of Restorative Dentistry, Ribeirão Preto, SP, Brazil.

**Keywords:** Dental Cements, Root Canal Irrigants, Dental Restoration, Temporary

## Abstract

Residual temporary cement may hinder fiber post bonding. Therefore, efficient root canal cleaning protocols are essential. This study investigated the effects of three cleaning methods on bond strength, adhesive interface quality as evaluated using scanning electron microscopy (SEM), and intratubular penetration of a self-adhesive resin cement as evaluated using confocal laser scanning microscopy (CLSM). Forty-two mandibular premolars were prepared using Reciproc R50, filled with AH Plus, and restored with provisional retainers cemented with Bifix Temp. Specimens were allocated to three groups (n = 14) according to the cleaning protocol: standardized drill (SD), drill with an ultrasonic insert (UI), or drill with a prophylactic brush (PB). Fiber posts were cemented using a self-adhesive resin cement. Push-out testing, failure mode analysis, and SEM/CLSM evaluations were performed. Bond strength (MPa) was analyzed using two-way ANOVA followed by Tukey's test, whereas failure patterns were analyzed using the chi-square test, and interface adaptation was analyzed using nonparametric tests. PB (4.42 ± 2.60 MPa) and UI (4.55 ± 2.41 MPa) showed significantly higher bond strength values and greater cement penetration than SD (3.04 ± 1.70 MPa) (p < 0.05). Mixed adhesive failures predominated, and SEM revealed no significant differences in interface adaptation. Cleaning with a PB or UI enhanced adhesive performance and resin cement penetration during fiberglass post cementation.

## Introduction

Anatomical complexity remains a significant challenge in the rehabilitation of teeth restored with fiberglass posts following endodontic treatment.^1^ This complexity may contribute to failure because of loss of adhesion.^2^ Consequently, cleaning dentinal surfaces—including the removal of debris,^3^ residual endodontic filling materials,^4^ and temporary adhesive cements^5,6^—remains a critical step and a significant clinical challenge.^7,8^ These procedures are essential for promoting optimal adaptation of resin-based materials and minimizing adverse effects on adhesive interface quality.^9^

Rehabilitation with fiberglass posts generally requires provisional restorations designed to restore form, function, and esthetics. Temporary cements are used with these restorations. However, the literature indicates that the chemical composition of temporary cements, particularly those containing eugenol,^10^ may negatively affect bond strength and adhesive interface quality between fiberglass posts and root dentin.^11^

Bifix Temp, a methacrylate-based temporary resin cement, provides improved esthetic properties and dual-curing capability. It is commonly used for temporary crown cementation.^12^ A previous study evaluated the effects of Bifix Temp on bond strength and adhesive interface quality between fiberglass posts and root dentin and found no adverse effects on either parameter, supporting its use as a superior alternative to eugenol-based and eugenol-free cements.^6^

Despite the favorable performance of temporary resin cements in the adhesive cementation of fiberglass posts, remnants of these materials, together with dentinal debris and endodontic filling materials, may persist on the root canal surface^6^ and thereby compromise resin cement penetration and adhesion. Mechanical protocols are therefore required to ensure more effective cleaning of the root dentin surface while minimizing additional dentin removal when drills are used.^13,14^ These protocols also aim to prevent gutta-percha plasticization and debris retention on root canal wall surfaces.^15^

Passive ultrasonic irrigation (PUI) uses acoustic energy transmitted from an insert to activate the irrigant within the root canal at frequencies ranging from 25 to 40 kHz. This method facilitates debris removal^16^ through acoustic micro-vaporization and enhances irrigant movement within the root canal system.^17^ PUI has been widely used to improve cleaning and disinfection in anatomically complex regions, such as isthmuses and lateral or accessory canals.^18^ It effectively reduces pulp tissue remnants, microbial load, and dentinal debris^19^ without adversely affecting resin cement polymerization.^20^ In addition, low-cost prophylactic root canal brushes have been introduced to assist in cleaning and removing dentinal shavings, the smear layer, and gutta-percha residues after drill preparation for post cementation, thereby improving resin cement adhesion.^13,14^ These brushes can be adapted to rotary systems (300–800 rpm) and aid in the removal of dentinal debris, necrotic tissue, and biofilm from root canal walls. They feature flexible, durable nylon filaments or synthetic bristles and are available in different sizes to accommodate variations in root canal diameter.

Although methacrylate-based temporary resin cements have been shown not to compromise adhesive interface quality or bond strength of fiberglass post systems^6^, comprehensive evidence regarding the effects of residual material remains limited. Therefore, this study aims to evaluate the effects of different root canal surface cleaning protocols on the bond strength of a self-adhesive resin cement and the adhesive interface quality between the intracanal restorative material and dentin. In addition, the study evaluates intratubular penetration of the resin cement using confocal laser scanning microscopy (CLSM) with fluorescence. The null hypothesis was that mechanical cleaning protocols using a prophylactic brush (PB) or ultrasonic insert (UI) would not significantly affect resin cement penetration, adhesive interface quality, or bond strength values.

## Methods

This study was approved by the Research Ethics Committee of the School of Dentistry of Ribeirão Preto, University de São Paulo (CAAE: 70776123.4.0000.5419).

Figure 1 presents a schematic flowchart illustrating the study design and methodology.

**Figure 1:**
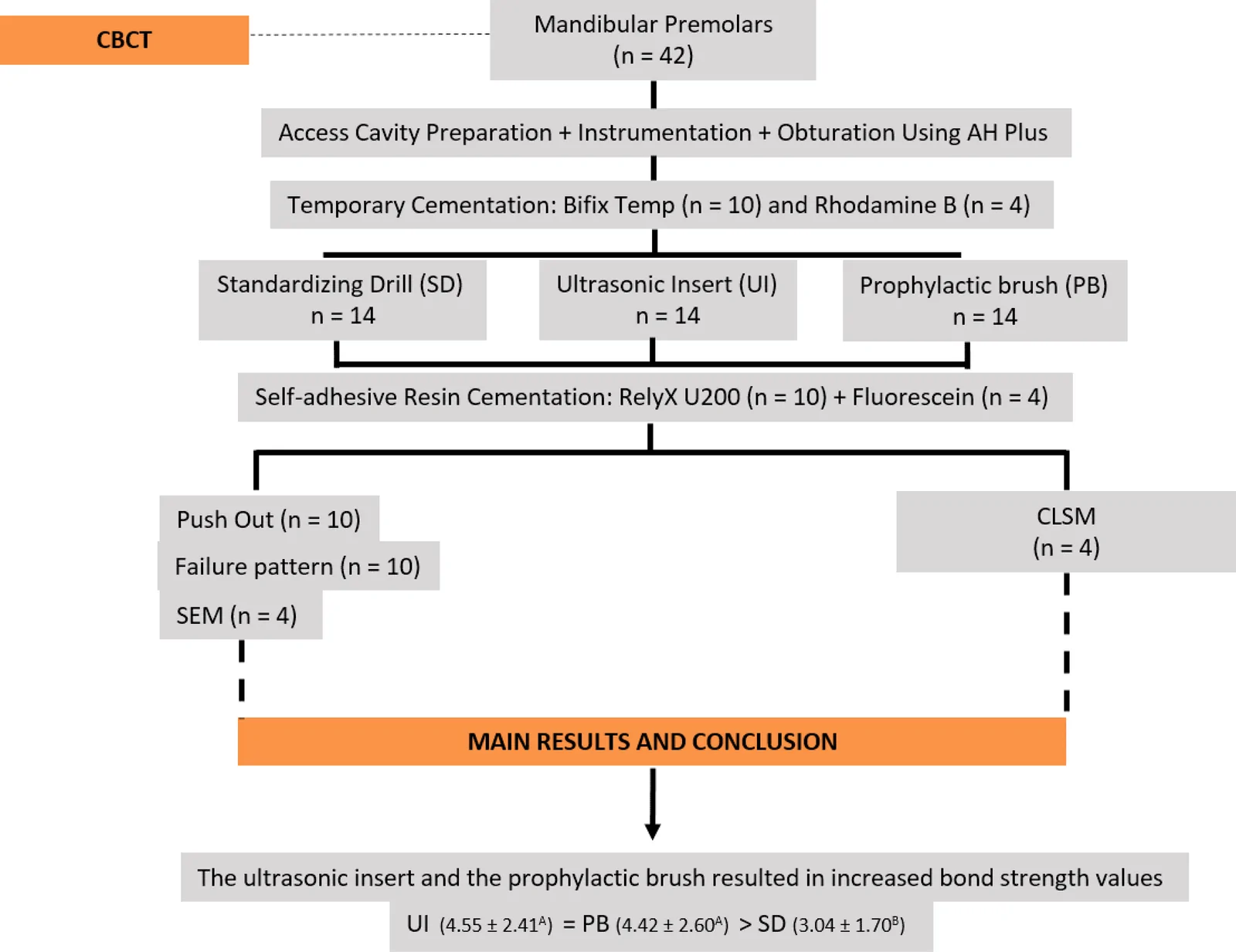
Schematic flowchart illustrating the study design and methodology.

### Selection and preparation of specimens

The sample size was calculated based on previous studies^6,9,19,21^ using a two-tailed test with a significance level of 5% (α = 0.05), 90% statistical power (1 − β = 0.90), an effect size of 0.94, and a 1:1 allocation ratio. The effect size of 0.94 was derived from previously reported bond strength data obtained under similar experimental conditions.^6,9,19,21^ The minimum required sample size was determined to be 14 specimens per group for bond strength testing, failure pattern analysis, adhesive interface evaluation, and CLSM analysis (SigmaPlot v.12.00, Systat Software, San Jose, USA).

Mandibular premolars with straight, fully developed roots and a minimum length of 14 mm were selected. The teeth were scanned using cone-beam computed tomography (CBCT) (OP300®, Prexion Co., Tokyo, Japan) under an endodontic acquisition protocol (90 kV, 4 mA, 37 s, isotropic voxel size of 0.10 mm, field of view: 6 × 4 mm).^6,9,19,21^ Morphometric data, including root canal circularity and major and minor diameters measured at 3.0 mm and 5.0 mm from the apical foramen, were obtained using OnDemand 3D Project Viewer software (Cybermed, Tustin, USA).^20^ Based on these measurements, 42 mandibular premolars with circular root canals (major-to-minor diameter ratio = 1.0 and roundness > 0.7) were selected.^20^

The crowns were sectioned at the cementoenamel junction using an Isomet 1000 cutting machine (Buehler, Lake Forest, USA) equipped with a 0.5 mm-thick diamond disc (South Bay Technology, San Clemente, USA). Sectioning was performed under refrigeration with a constant load of 75 g at a speed of 350 rpm, standardizing root length to 14 mm.

### Sample preparation

Root canals were initially irrigated with 2 mL of 2.5% sodium hypochlorite (NaOCl) using a disposable plastic syringe (Ultradent Products, South Jordan, USA) and a 0.30 mm NaviTip needle (Ultradent Products, South Jordan, USA). Foraminal patency was confirmed with a #15 stainless steel K-type hand file (Dentsply Maillefer, Petrópolis, Brazil), which was passively advanced until the tip became visible at the apical foramen, thereby determining actual tooth length. From this measurement, 0.5 mm was subtracted to establish the working length (WL).

Teeth with a standardized apical diameter of 300 μm, confirmed using a #30 K-file (Dentsply Maillefer, Petrópolis, Brazil), were selected. Biomechanical preparation was carried out using the crown-down technique with the Reciproc R50 instrument (VDW, Munich, Germany), operated with a VDW Silver electric motor (Reciproc, VDW, Munich, Germany). For each millimeter of advancement into the canal, it was irrigated with 5 mL of 2.5% NaOCl. After instrumentation, final irrigation was performed using 2 mL of 17% ethylenediaminetetraacetic acid (EDTA) for 5 min, followed by 5 mL of 2.5% NaOCl. Drying was performed with a Capillary Tip suction cannula (Ultradent Products, South Jordan, USA), followed by R50 absorbent paper cones (Reciproc, VDW, Munich, Germany).

The R50 gutta-percha cone (Reciproc, VDW, Munich, Germany) was fitted to the WL and confirmed using orthoradial and mesioradial digital radiographs obtained with a digital sensor (Fona CDRelite, Schick, DMM, Bandeirantes, Brazil). Obturation was performed using the single-cone technique. AH Plus root canal sealer (Dentsply DeTrey, Konstanz, Germany) was mixed according to the manufacturer's instructions and applied to the root canal using a #40 K-file (Dentsply Maillefer, Petrópolis, Brazil) with counterclockwise rotation. The main cone was coated with root canal sealer and inserted into the canal with gentle rotational movements up to the WL. Gutta-percha was trimmed using a Schilder condenser (Thimon, São Paulo, Brazil) heated in a flame, followed by vertical condensation in the cold state after each removal of excess material until a 4-mm apical plug of filling material remained. The canal was cleaned with cotton soaked in 90% alcohol to remove residual sealer, and obturation quality was confirmed using orthoradial and mesioradial digital radiographs. Specimens were stored in numbered Eppendorf tubes at 100% relative humidity at 37°C for 72 h, corresponding to three times the sealer setting time.

After excess gutta-percha was trimmed, the roots were prepared for provisional post space. Post spaces were prepared to a depth of 10 mm using the Exacto No. 1 drill, corresponding to the diameter of the Exacto #1 post (Angelus, Londrina, Brazil). Drills were replaced after every five preparations. The canals were irrigated with 5 mL of distilled water and dried with Capillary Tip suction cannulas.

### Fabrication of provisional intraradicular retainers

Provisional intraradicular retainers were fabricated using orthodontic wire and Z350 composite resin (3M ESPE, St Paul, USA) to ensure optimal adaptation to the root canal walls. Initially, a layer of liquid glycerin (Farmax, Divinópolis, Brazil)—a water-soluble gel—was applied to the root canal walls using a microbrush (3M ESPE, St. Paul, USA) to prevent resin adhesion. Microretentions were created on the orthodontic wire using a 0.6 mm-thick Carborundum disc (American Burrs, Palhoça, Brazil) to enhance resin adhesion to the wire. The wire–resin assembly was then positioned inside the root canal.

Continuous photoactivation was performed for 5 s using a Dabi Atlante Optilight Max photopolymerizer (Alliage, Ribeirão Preto, Brazil), operating at a wavelength of 420–500 nm and a power of approximately 1150 mW/cm², with light intensity verified using a digital radiometer (Optical Power Meter, 1830C, Newport, USA). This was followed by an additional 20 s of photoactivation performed outside the root canal. The canal was then irrigated with 5 mL of distilled water to remove the glycerin, aspirated with a suction cannula, and dried with R50 absorbent paper points (Reciproc, VDW, Munich, Germany).

The temporary cement Bifix Temp—a methacrylate-based resin material (VOCO, Cuxhaven, Germany) (Table 1) —was prepared by mixing equal parts of base and catalyst on a glass slab according to the manufacturer's instructions. The provisional retainers were coated with the cement and inserted into the root canal (n = 10).

**Table 1:** Brand name, composition, manufacturer, and batch number according to the manufacturer's information.

Material	Composition	Manufacturer	Batch No.
Bifix Temp	Base paste: dimethacrylates (UDMA, TEGDMA, PEGDMA), amine, camphorquinone (CQ), pyrogenic silica.	VOCO, Cuxhaven, Germany	
	Catalyst paste: dimethacrylates (UDMA, TEGDMA, PEGDMA), benzoyl peroxide (BPO), butylated hydroxytoluene (BHT), pyrogenic silica.		2246631
RelyX U200	Base paste: silane-treated glass powder, 2-propenoic acid, 2-methyl-, 1,1’-[1-(hydroxymethyl)-1,2-ethanediyl] ester, triethylene glycol dimethacrylate (TEGDMA), silane-treated silica, glass fibers, sodium persulfate, and tert-butyl per-3,5,5-trimethylhexanoate.	3M ESPE, St. Paul, SA	
	Catalyst paste: silane-treated glass powder, substituted dimethacrylate, silane-treated silica, sodium p-toluenesulfinate, 1-benzyl-5-phenylbarbituric acid, calcium salts, 1,12-dodecane dimethacrylate, calcium hydroxide, and titanium dioxide (filler content ≅ 70%)		10769525

### Cleaning the root canal and cementing the fiberglass posts

Roots with provisional retainers were stored for 7 days in an incubator at 37°C and 100% relative humidity. Provisional retainers were manually removed using Kelly hemostatic forceps (Golgran, São Caetano do Sul, Brazil). The roots were randomly assigned to three groups by a single operator who performed all procedures. Group allocation was based on the cleaning method used to remove residual provisional cement before fiberglass post cementation: a) standardized drill (SD), b) standardized drill plus UI, and c) standardized drill plus PB.

An operator blinded to the study objectives generated a randomization list using the Sealed Envelope website (https://www.sealedenvelope.com/) with a 1:1 allocation ratio and random block sizes. The allocation list was stored securely and accessed only by a blinded assistant after specimen inclusion. Based on this list, each tooth was assigned an identification number and randomly allocated to a group.

In the SD group, residual temporary cement was removed using the same standardized drill (Exacto No. 1) employed for post space preparation, up to 4 mm short of the apical end of the gutta-percha. In the other groups, cleaning followed the same procedure as the SD group and was supplemented with either UI or PB up to 4 mm apical to the gutta-percha. In the UI group, a Flatsonic insert (Helse Ultrasonic, Santa Rosa de Viterbo, Brazil) was attached to the ultrasonic device (Advance 1, Microdont, São Paulo, Brazil) operated at 30% power with an open irrigation system for 30 s. In the PB group, the brush was coupled to a low-speed handpiece and used without water cooling. The canals were then irrigated with 5 mL of distilled water, aspirated using Capillary Tip suction cannulas, and dried with R50 absorbent paper points (Reciproc, VDW, Munich, Germany).

The roots were wrapped with wax to prevent additional polymerization of the composite resin and resin cement. The fiberglass posts were positioned in the root canals to confirm a standardized depth of 10 mm before being trimmed coronally using a tapered diamond bur, ensuring that no more than 4 mm extended beyond the canal. This adjustment allowed for more effective light curing across all root thirds. Prior to cementation, the posts were anatomically shaped to minimize the gap between the post surface and canal walls. The posts were immersed in 70° GL ethanol for 10 min, air-dried, and treated with a silane coupling agent (Angelus, Londrina, Brazil) applied with a microbrush. The silane was allowed to react for 60 s and then was gently air-dried. Subsequently, a universal adhesive (3M ESPE, St. Paul, USA) was applied to the post surface with a microbrush and light-cured for 20 s.

A layer of liquid glycerin was applied to the root canal walls using a microbrush to prevent resin adhesion to the dentinal walls. Z350 composite resin was applied around the fiberglass post to improve adaptation to the canal walls. The post-resin assembly was then inserted into the canal and photoactivated for 5 s. It was subsequently removed and subjected to an additional 20 s of photoactivation.

The root canal was irrigated with distilled water to remove the water-soluble gel from the canal walls. The surface of the anatomically individualized post was prepared following the previously described protocol. The self-adhesive resin cement RelyX U200 (3M ESPE, St. Paul, USA) (Table 1) was prepared according to the manufacturer's instructions and inserted into the root canal using a #50 K-file. The cement was also applied to the post surface, and the assemblies were inserted to a depth of approximately 10 mm under digital pressure. Excess material was removed with a #5 exploratory probe (Golgran, São Caetano do Sul, Brazil) to ensure proper sealing at the cervical region of the root.

Photoactivation was performed for 40 s using a Dabi Atlante Optilight photopolymerizer, with the curing light tip placed in direct contact with the coronal end of the post. The canal orifice was then sealed with Z350 composite resin (3M ESPE, St. Paul, USA), and the specimens were stored in an incubator at 37 °C for 7 days. All biomechanical preparation procedures were performed by a single operator with 10 years of experience under ambient lighting to ensure consistency among specimens. The operator performed obturation, canal preparation, and post cementation.

### Sectioning of specimens

After 14 days of cementation, the specimens were positioned on acrylic resin plates with their longitudinal axis parallel to the surface and fixed using hot glue. This configuration ensured that the roots were sectioned perpendicularly to the long axis in the mesiodistal direction. Sectioning was performed using a 0.3 mm-thick diamond disc (South Bay Technology, San Clemente, USA) under constant cooling at a speed of 350 rpm and a load of 75 g, using an Isomet 1000 precision cutting machine.

Two dentin slices (1.0 ± 0.1 mm thick) were obtained from each root third and slice thicknesses were confirmed using a digital caliper (Digimess, Shiko Precision Gaging, China). The most cervical slices from each third were allocated to the push-out test, followed by failure pattern analysis (n = 10). The most apical slices of each third were allocated to adhesive interface evaluation using scanning electron microscopy (SEM) (n = 4).

### Push-out bond strength test

The slices were positioned on stainless steel bases attached to the lower component of an Instron 2519-106 universal testing machine (Instron, Canton, USA). These bases featured central holes with diameters of 2.5 mm, 1.5 mm, and 1.2 mm to accommodate slices of different sizes. Each specimen was aligned with the corresponding hole in the base, with the cervical side facing downward. Metal rods with active tip diameters of 1.5 mm, 1.0 mm, and 0.8 mm were used for the cervical, middle, and apical thirds, respectively. These rods were attached to the upper component of the testing machine and positioned over the intracanal restorative material. The test was conducted at a constant speed of 0.5 mm/min until failure occurred under maximum load.

The force required for displacement was recorded in Newtons (N). Bond strength was calculated in megapascals (MPa) by dividing the force by the lateral area of the intracanal restorative material.^22^ The bonded lateral area (SL) was calculated based on the geometric characteristics of the intracanal restorative material (resin cement + fiberglass post) at the level of each section.

Slice height (h) was measured using a digital caliper (Digimess, Shiko Precision Gaging, China), and the largest and smallest radii were measured using a stereomicroscope (Leica M165C, Leica Microsystems, Germany) with LAS v4.4 software (Leica Microsystems, Germany).

The cement adhesion area (SL), expressed in mm², was calculated using the following formula for the lateral area:


$${\text{S}}_{\text{L}}=\text{π}\left(\text{R}+\text{r}\right)\sqrt{{\text{h}}^{2}+{\left(\text{R}-\text{r}\right)}^{2}}$$

Where:

R: radius of the restorative material at the coronal portion; r: radius at the apical portion; h: height or thickness of the slice.

Bond strength (BS), expressed in MPa, was determined by dividing the force (F) required to displace the fiberglass post by the calculated lateral area (SL), as follows: BS = F / SL.

### Failure pattern analysis

The slices were examined to determine failure patterns using a Leica M165C stereomicroscope (Leica Microsystems, Mannheim, Germany) with LAS v4.4 software (Leica Microsystems, Mannheim, Germany) at 25× magnification. Failure patterns were expressed as percentages and classified as: a) adhesive to dentin, in which the restorative material detached from dentin; b) adhesive to the resin cement, in which the fiberglass post detached from the resin cement; c) mixed, involving detachment from both dentin and resin cement; d) cohesive in dentin, involving dentin fracture; and e) cohesive in the fiberglass post, involving fracture of the post (Figure 2).

**Figure 2:**
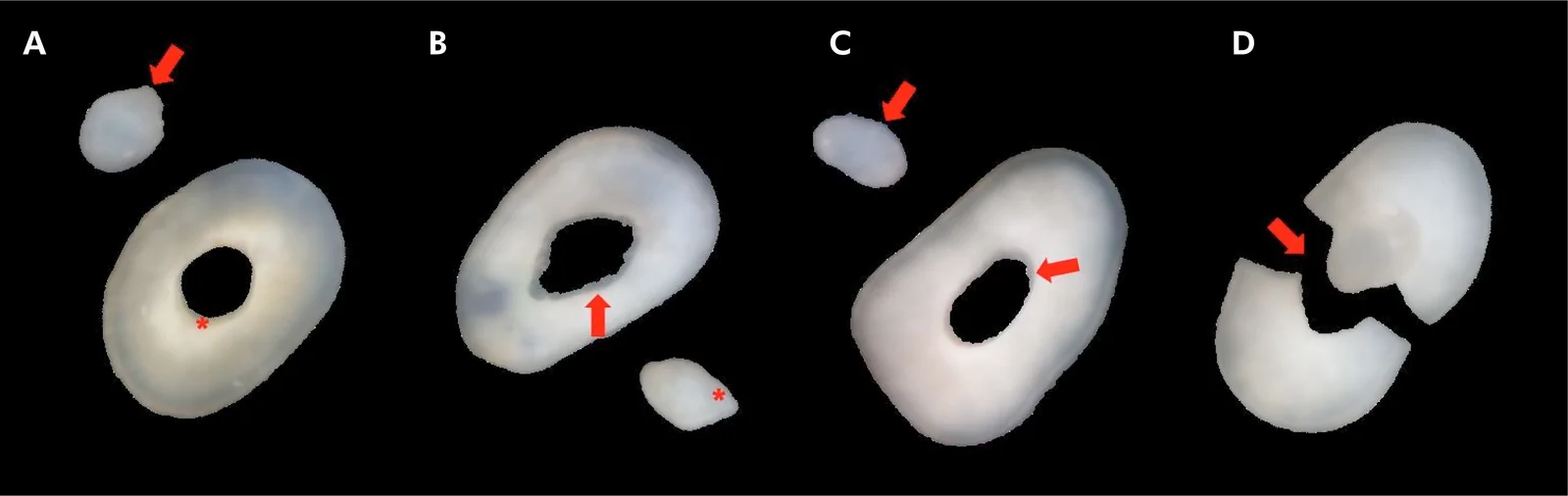
Representative images of the failure patterns observed after the bond strength test.

(A) Adhesive to dentin, showing absence of resin cement on dentin walls (red asterisk). (B) Adhesive to the post, showing resin cement on dentin walls (red arrow). (C) Mixed failure, showing resin cement on dentin walls (red arrow) and around the fiberglass post (red arrow). (D) Cohesive in dentin, showing dentin fracture (red arrow).

Prior to failure pattern analysis, two examiners were calibrated using 10 pilot samples. Each examiner independently classified the failure modes, and disagreements were resolved through discussion until consensus was reached. The calibration process was repeated until an inter-examiner agreement of at least 90% was achieved, ensuring consistency throughout the study.

### Analysis of the adhesive interface using SEM

Dentin slices were sequentially polished with #400-, #600-, and #1200-grit waterproof abrasive paper, followed by 1.0 μm and 0.3 μm alumina pastes (Arotec, São Paulo, Brazil) under constant cooling using a felt disk and polishing machine (Arotec APL-4). The slices were then placed in plastic holders for immersion and solution exchange during subsequent steps.^6,9^

The slices were rinsed with running water, cleaned in an ultrasonic bath (Ultrasonic Cleaner 1440D, Odontobrás, Ribeirão Preto, Brazil) with deionized water for 10 min, superficially decalcified in 6 M hydrochloric acid for 30 s, and deproteinized in 2% NaOCl for 10 min. After rinsing, specimens were fixed with 2.5% glutaraldehyde buffered with 0.1 M sodium cacodylate (pH 7.4) for 12 h at 4°C, followed by immersion in sodium cacodylate for 1 h, with three buffer exchanges. Specimens were dehydrated in an ascending ethanol series (25%, 50%, 75%, 95% for 20 min each, followed by 100% ethanol for 1 h) and treated with hexamethyldisilazane (HMDS, Ted Pella, Redding, USA) for 10 min under a fume hood.^6,9^

After dehydration, specimens were mounted on metal stubs with double-sided adhesive tape (Electron Microscopy Sciences, Washington, DC, USA) and coated with a 30 nm gold–palladium layer using a Desk II Denton Vacuum sputter coater. SEM analysis was performed using a JSM-6610LV SEM (JEOL, Akishima, Japan) at 20 kV with SEM Control User Interface v.3.06 software. Photomicrographs were obtained at 40× and 80× magnifications. At 80× magnification, twelve equidistant measurements were obtained along the adhesive interface to identify voids (gaps).^6,9^

Cement adaptation to the root canal wall was classified according to previous studies^6,9,23^ as: a) good, when most sections showed no gaps between the cement and dentin; b) fair, when most sections showed small gaps (< 1 μm); c) poor, when most sections showed multiple gaps (1–10 μm); and (4) no adaptation, when most sections showed gaps > 10 μm.

### Penetration analysis using CLSM

Provisional root retainers were fabricated, and four specimens per group were cemented with Bifix Temp resin cement prepared according to the manufacturer's instructions and mixed with 0.1% fluorescein dye (Sigma-Aldrich, Merck KGaA, Darmstadt, Germany). The provisional posts were coated, inserted into the root canals, photoactivated for 40 s, and the specimens were stored at 37°C for 7 days.

After 7 days, the temporary retainers were removed, and canal cleaning protocols specific to each group were performed. RelyX U200 resin cement was prepared according to the manufacturer's instructions and mixed with 0.1% rhodamine B dye (Sigma-Aldrich, Merck KGaA, Darmstadt, Germany). Fiberglass posts were cemented following the previously described procedures. The roots were then stored at 37°C for an additional 7 days before sectioning.

The roots were mounted on acrylic resin plates and sectioned into 1.0 mm-thick slices. The most apical slices of each third were selected for qualitative analysis (n = 4). Images of the dentin/restorative material interface were obtained at 100× magnification using a Leica CLSM with a 5 × 5 mm field of view, 512 × 512 pixel resolution, and excitation/emission wavelengths of 553/568 nm (rhodamine B) and 360/449 nm (fluorescein).^24^ The system was calibrated before each session using a fluorescent standard. Epifluorescence mode was used to analyze images to a depth of 10 μm to evaluate material penetration into dentinal tubules. All images were evaluated twice by a single blinded operator to ensure CLSM measurement reproducibility. The intraclass correlation coefficient (ICC) was calculated, and ICC values ≥ 0.90 indicated excellent intra-examiner reliability.

### Statistical analysis

Bond strength data were presented as mean values and standard deviations. Normality was assessed using the Shapiro–Wilk test (p > 0.05), and homogeneity of variance was evaluated using Levene's test (p > 0.05). Because the data met these assumptions, comparisons between groups were performed using two-way ANOVA followed by Tukey's post hoc test to evaluate the effects of the factors "cleaning protocol" and "root third."

The chi-square test was used to assess associations between failure patterns and the factors "cleaning protocol" and "root third." Data on cement adaptation to the root dentin wall were analyzed using the Kruskal–Wallis test followed by the Dwass–Steel–Critchlow–Fligner post hoc test. All statistical analyses were performed using Jamovi v.1.6.23 (The Jamovi Project, Sydney, Australia), with a significance level of 5%.

## Results

Two-way ANOVA revealed significant effects of the factors "cleaning protocol" (p < 0.001) and "root third" (p < 0.001). Regarding the cleaning protocol, both the PB and UI showed significantly higher bond strength values than SD (p = 0.005 and p = 0.002, respectively), with no statistically significant difference between them (p = 0.968) (Table 2). Figure 3 shows that the PB and UI groups exhibited higher median bond strength values than the drill group.

**Table 2:** Bond strength values expressed as mean ± standard deviation, in megapascals (MPa), and 95% confidence intervals (CI) of the restorative material–dentin interface for the different root canal cleaning protocols.

Cleaning protocol	Bond strength (MPa)	95%CI
SD	3.04 ± 1.70^B^	2.54–3.53
PB	4.42 ± 2.60^A^	3.66–5.18
UI	4.55 ± 2.41^A^	3.87–5.22

SD: standardized drill; PB: prophylactic brush; UI: ultrasonic insert. Different uppercase letters indicate statistically significant differences according to Tukey's post hoc test (p < 0.05).

**Figure 3:**
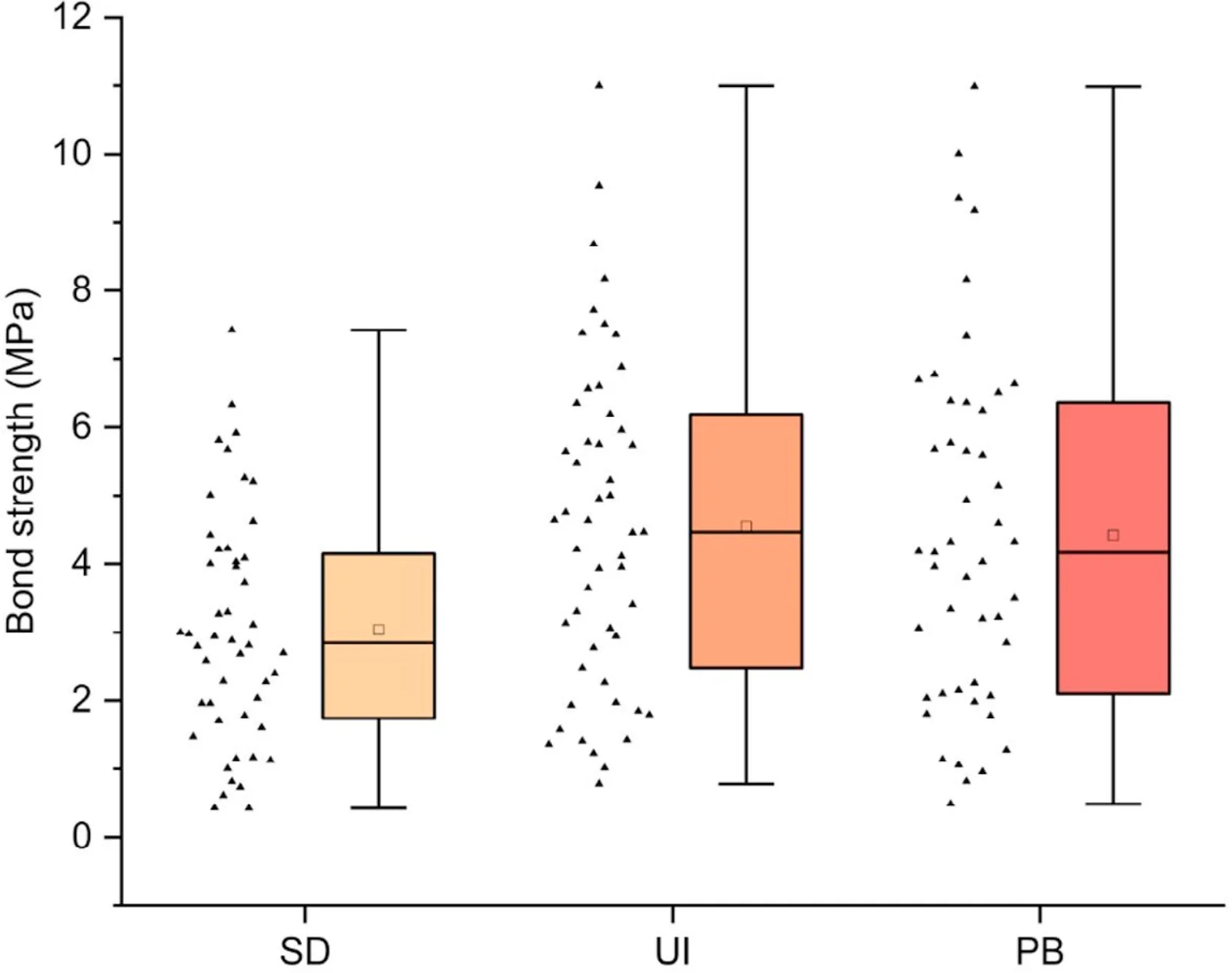
Distribution of bond strength values according to the cleaning protocol.

SD: standardized drill; UI: ultrasonic insert; PB: prophylactic brush.

Analysis of the root third factor showed that the cervical third had higher bond strength values than the middle and apical thirds (p < 0.001), with no statistically significant difference between the middle and apical thirds (p = 0.093) (Table 3). Figure 4 shows that the cervical third exhibited higher median bond strength values in the cervical third.

**Table 3:** Bond strength values expressed as mean ± standard deviation (MPa) and 95% confidence intervals (CI) of the restorative material–dentin interface according to the root third.

Root third	Bond strength	95%CI
Cervical	5.38 ± 2.38^A^	4.71–6.04
Middle	3.69 ± 1.71^B^	3.20–4.17
Apical	2.74 ± 2.16^B^	2.08–3.41

Different uppercase letters indicate statistically significant differences according to Tukey's post hoc test (p < 0.05).

**Figure 4:**
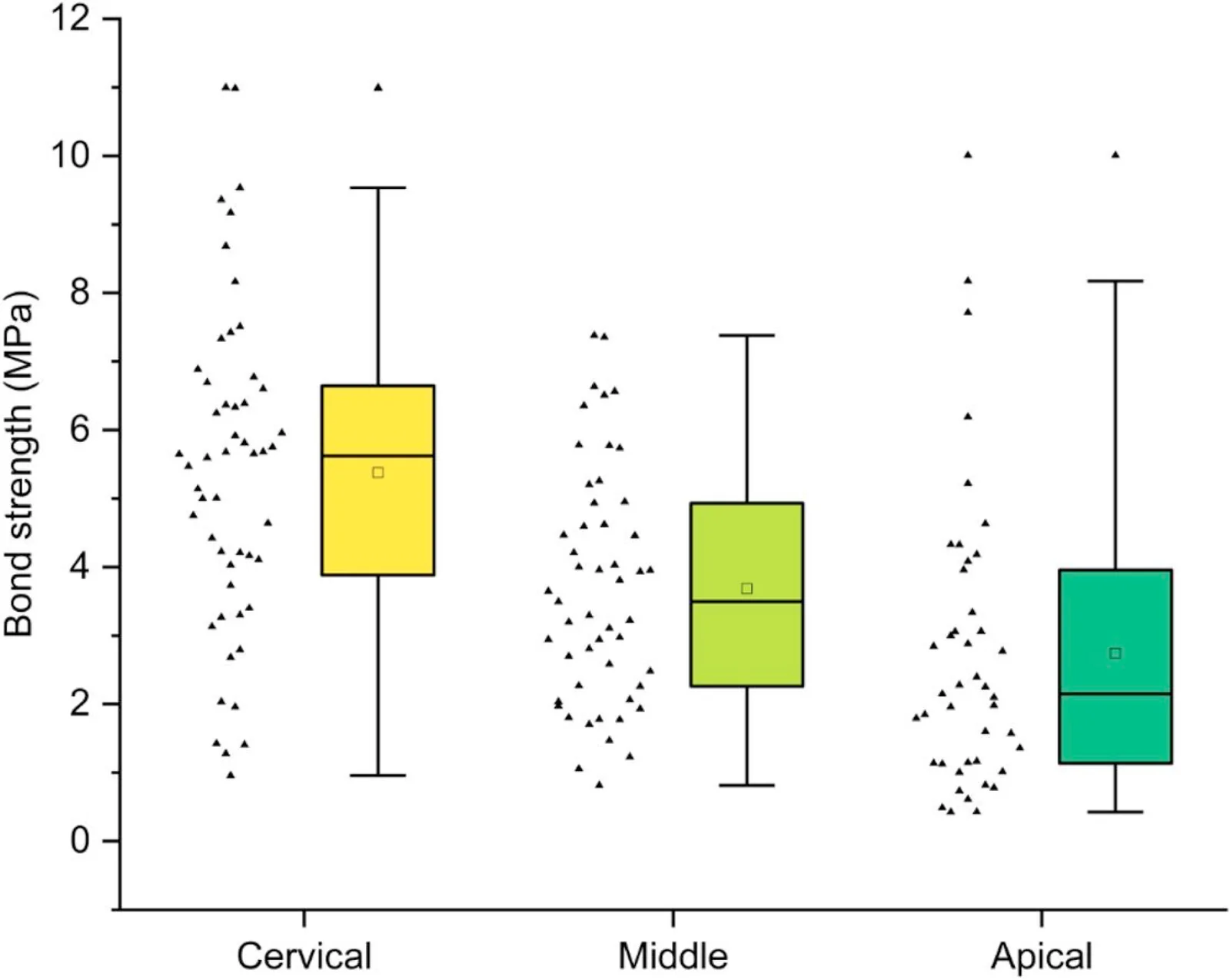
Distribution of bond strength values according to root third.

The chi-square test showed no significant association between the failure pattern and the factors "cleaning protocol" (p = 0.942) and "root third" (p = 0.066). Failure pattern data are presented as percentages in Table 4.

**Table 4:** Failure patterns after the push-out test according to the root canal cleaning protocols (percentage values).

Cleaning protocol	Failure pattern	Root third		
		Cervical	Middle	Apical
SD	Da	20.00	23.50	26.66
	Af	0.00	23.50	0.00
	Ma	80.00	47.05	66.66
	Dc	0.00	5.88	6.68
PB	Da	29.41	20.00	26.66
	Af	17.64	6.66	0.00
	Ma	52.94	73.33	53.33
	Dc	0.00	0.00	20.00
UI	Da	26.31	15.78	7.69
	Af	10.52	5.26	0.00
	Ma	63.15	68.42	69.23
	Dc	0.00	10.52	23.07

SD: standardized drill; PB: prophylactic brush; UI: ultrasonic insert; Da: adhesive to dentin; Af: adhesive to the fiberglass post; Ma: mixed adhesive failure; Dc: cohesive in dentin.

The Kruskal–Wallis test showed no statistically significant differences in adhesive interface adaptation between the factors "cleaning protocol" (SD, PB, and UI) (p = 0.320) and "root third" (cervical, middle, and apical) (p = 0.659). These data are presented as percentages in Table 5.

**Table 5:** Percentage distribution and 95% confidence intervals (CI) of restorative material adaptation according to the root canal cleaning protocols. Adaptation criteria to root dentin: (1) good; (2) fair; (3) poor; (4) no adaptation.

Score	Cleaning protocol		
	SD % (95%CI)	PB % (95%CI)	UI % (95%CI)
1	41.70 (21.90–61.50)	33.30 (14.00–52.00)	16.70 (0.00–30.90)
2	0.00 (0.00–14.00)	0.00 (0.00–14.00)	0.00 (0.00–14.00)
3	50.00 (29.90–70.10)	50.00 (29.90–70.10)	58.30 (38.30–78.10)
4	8.30 (0.00–19.90)	16.70 (0.00–30.90)	25.00 (7.70–42.30)

SD: standardized drill; PB: prophylactic brush; UI: ultrasonic insert.

Qualitative analysis of CLSM images showed that, after all cleaning protocols were applied, the temporary cement Bifix Temp was observed within dentinal tubules, forming short tags that were more evident in the middle and apical thirds. Interaction with the resin cement RelyX U200, visualized as a yellow-orange fluorescent signal (Figure 5), was also observed. This interaction did not affect adhesive interface quality, indicating adequate adaptation between the fiberglass post and root dentin.

**Figure 5:**
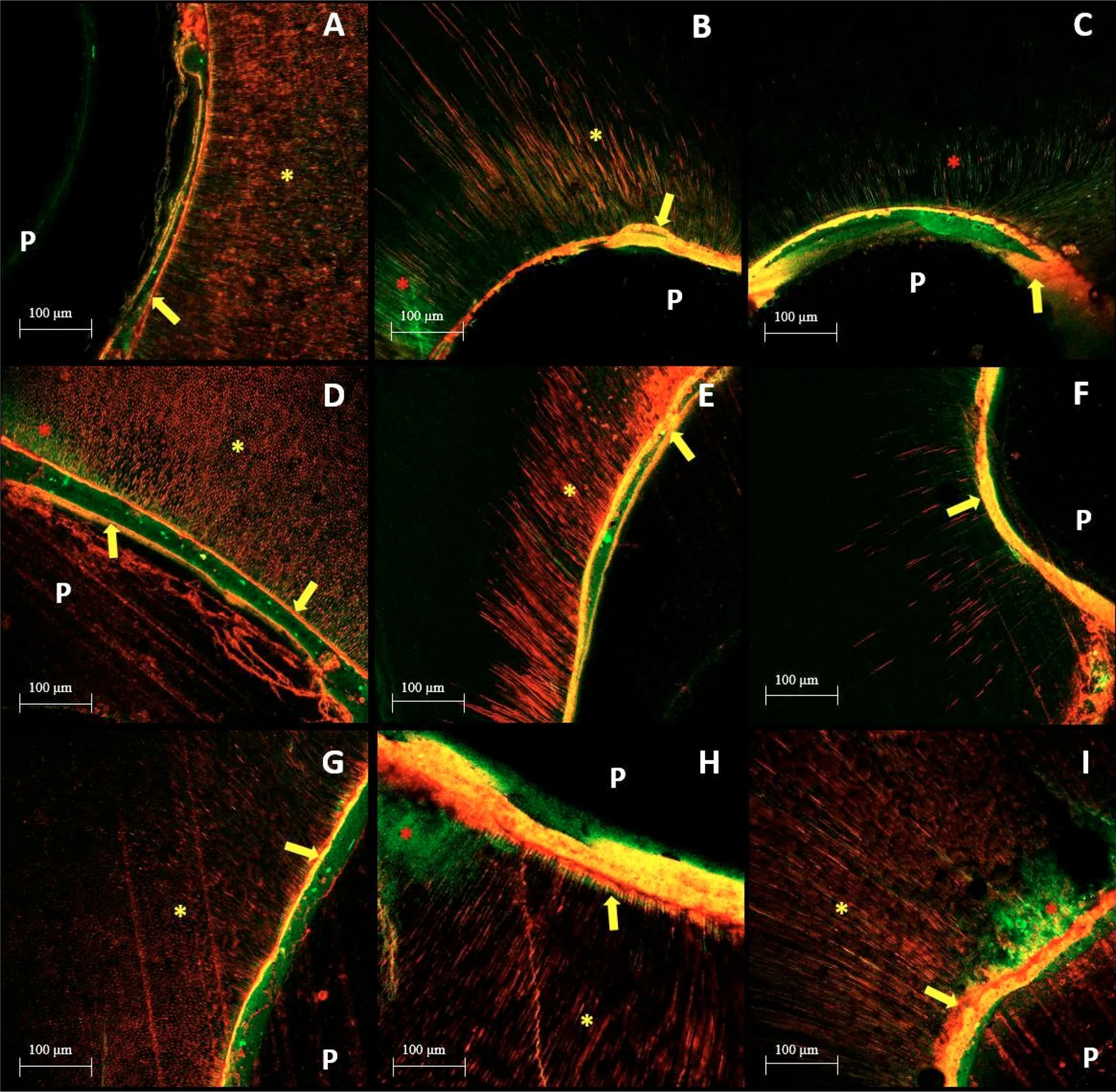
Confocal laser scanning microscopy (CLSM) images showing intratubular penetration of the self-adhesive resin cement RelyX U200 after provisional post cementation and application of different root canal cleaning protocols.

(A–C) Intratubular penetration after root canal cleaning with the standardized drill in the cervical, middle, and apical thirds, respectively; (D–F) Intratubular penetration after root canal cleaning with the prophylactic brush in the cervical, middle, and apical thirds, respectively; (G–I) Intratubular penetration after root canal cleaning with the ultrasonic insert in the cervical, middle, and apical thirds, respectively. p: post; Yellow asterisks: intratubular penetration of RelyX U200 self-adhesive resin cement stained with rhodamine B. Red asterisks: intratubular penetration of the temporary cement Bifix Temp stained with fluorescein. Yellow arrows: interaction between Bifix Temp and RelyX U200 at the adhesive interface.

Figure 5 shows that the highest penetration of the self-adhesive resin cement RelyX U200 into dentinal tubules, characterized by long tags, was observed by CLSM in the cervical third, followed by the middle and apical thirds, regardless of the cleaning protocol used. In the drill group, the middle and apical thirds (Figures 5A and 5B) exhibited lower intratubular penetration with shorter tags compared with the other groups (Figures 5A–5F, 5H, 5I).

The percentage distribution of restorative material adaptation according to the root thirds is showed in Table 6. Qualitative analysis of SEM images (Figure 6) showed greater maladaptation at the adhesive interface between the fiberglass post, resin cement, and root dentin in the drill group, as indicated by yellow arrows (Figures 6A–6C). In contrast, enhanced adaptation was observed in the PB (Figures 6D–6F) and UI (Figures 6G–6I) groups, particularly in the middle and apical thirds, as indicated by yellow asterisks.

**Table 6:** Percentage distribution of restorative material adaptation according to the root thirds (percentage values).

Score	Root third		
	Cervical	Middle	Apical
1	25.00	25.00	41.70
2	0.00	0.00	0.00
3	50.00	66.70	41.70
4	25.00	8.30	16.70

**Figure 6:**
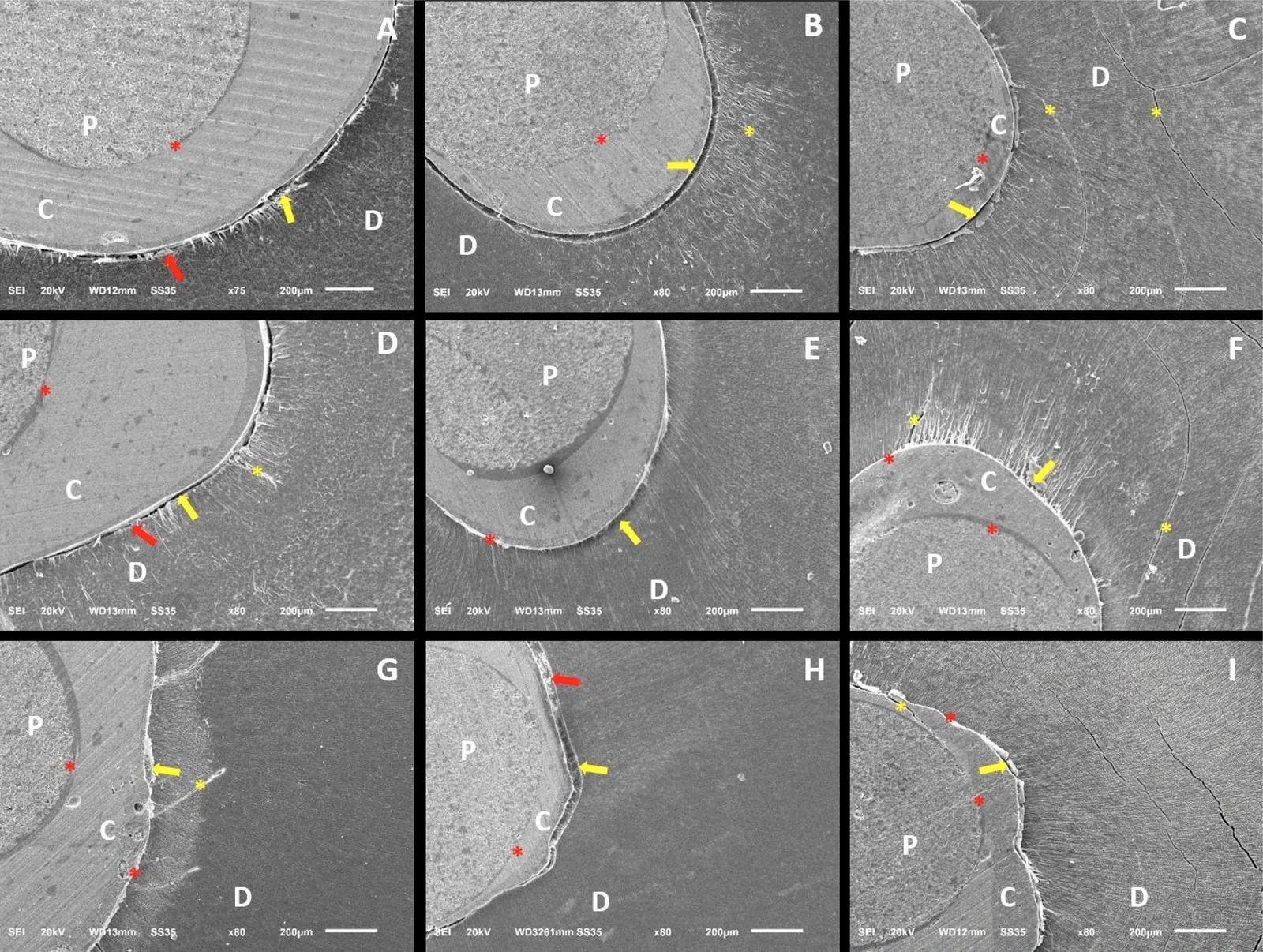
Scanning electron microscopy (SEM) photomicrographs of the adhesive interface between the fiberglass post, RelyX U200 self-adhesive resin cement, and root dentin after application of different root canal cleaning protocols at 80× magnification.

(A–C) Adhesive interface after root canal cleaning with the standardized drill in the cervical, middle, and apical thirds, respectively. (D–F) Adhesive interface after root canal cleaning with the prophylactic brush in the cervical, middle, and apical thirds, respectively. (G–I) Adhesive interface after root canal cleaning with the ultrasonic insert in the cervical, middle, and apical thirds, respectively. p: post. c: resin cement. d: dentin. Yellow arrows: gaps at the adhesive interface. Red arrows: remnants of temporary cement. Yellow asterisks: microfractures. Red asterisks: juxtaposition at the adhesive interface.

## Discussion

Loss of adhesion is one of the primary causes of failure in treatments with fiberglass posts,^25,26^ often attributed to residual materials adhering to the root canal walls.^3,11^ This study demonstrated that mechanical cleaning protocols using UI or PB significantly enhanced the penetration of self-adhesive resin cement into dentinal tubules and increased bond strength, thereby rejecting the null hypothesis. However, the protocols evaluated did not produce a statistically significant effect on the adhesive interface quality, corroborating the null hypothesis in this respect.

All fiberglass posts were anatomically customized to reduce cement layer thickness and improve adaptation to the root canal walls, thereby promoting more effective interaction with dentin.^9,27^ The use of a self-adhesive resin cement facilitated clinical handling due to lower viscosity compared with that of universal adhesive resin cements. This property not only enhanced the flow and adaptation of the material, especially in narrower regions of the canal, but also contributed to improving chemical bonding with hydroxyapatite in the dentin.^9,28^ Such bonding is particularly relevant in the middle and apical thirds, where access is limited and stable hybrid layer formation is more challenging.

The push-out test was selected to evaluate bond strength because it allows direct application of force to the adhesive interface, thereby reducing stress concentration and minimizing contact with the surrounding dentin. This method provides more accurate and reliable measurements while better simulating the clinical conditions of post cementation.^29,30^ Failure patterns were analyzed using stereomicroscopy, whereas SEM images were used to evaluate gaps at the adhesive interface.^31,32^ These gaps were classified by quadrant and examined at multiple magnifications using high-resolution imaging^23,33^.

Analysis of CLSM images showed that the UI and PB protocols increased resin cement penetration into dentinal tubules across all root thirds, suggesting effective removal of debris and residual materials from the root canal surface.^34,35^ In contrast, SEM analysis showed no significant differences in adhesive interface quality between groups, thereby supporting the null hypothesis for this outcome. However, CLSM images demonstrated the presence of residual Bifix Temp cement within dentinal tubules, which may have chemically interacted with RelyX U200, preserving the collagen fiber structure and facilitating diffusion of acid monomers that anchored the polymer network.^12^ This interaction did not compromise adhesive interface quality, aligning with previous findings.^6^

The ultrasonic insert passively enhanced cleaning efficacy without modifying the dentin substrate,^14,35^ through acoustic streaming and cavitation, displacing residual materials and debris from the deeper regions of the root canal.^13,14,35^ Similarly, the PB effectively displaced particles by bristle friction, thereby showing results comparable with those of the UI in both resin cement penetration and bond strength.^13,14^ These additional cleaning and surface preparation methods for root canal walls are consistent with similar studies, where complementary techniques resulted in reduced residual debris and increased bond strength.^36,37^

Bond strength and resin cement penetration were highest in the cervical third, attributed to better light proximity for photopolymerization and larger dentinal tubule diameters facilitating cleaning.^9,28^ The improved penetration of adhesive cements may be attributed to increased surface roughness and the formation of resin tags within dentinal tubules. Mechanical or ultrasonic cleaning methods can expose the underlying dentin and create micro-retentive conditions, facilitating deeper infiltration of the adhesive and enhancing micromechanical interlocking, which ultimately contributes to higher bond strength.^9,28^ Nonetheless, the UI and PB protocols demonstrated significantly greater resin cement penetration and bond strength than the SD protocol. These findings suggest that adjunctive cleaning techniques are essential in clinical practice to improve the quality of adhesion and reduce the risk of bonding failures following endodontic treatment and fiberglass post cementation.

The use of ultrasonic inserts and prophylactic brushes for root canal cleaning increased bond strength. These findings highlight the importance of implementing such protocols before fiberglass post cementation in restorative treatments, because adhesion failure remains one of the primary causes of treatment failure.

The middle and apical thirds demonstrated lower bond strength and penetration due to limited light access, reduced cleaning efficiency, and slower chemical polymerization, which produced weaker bonds.^38^ Studies have investigated the effect of light-curing distance on push-out bond strength and have observed that increased distance reduced bond strength in the apical region by decreasing the degree of polymerization, thereby resulting in weaker cross-linking between the resin cement and root dentin.^39^

A meta-analysis^40^ investigated strategies for minimizing the adverse effects of temporary cementation and evaluated the influence of different temporary cements and their removal methods on bond strength. Because temporary cement residues may impair the wetting and infiltration capacity of luting cements, complete removal is necessary before undertaking subsequent adhesive procedures. The analysis demonstrated that resin-based polycarboxylate and calcium hydroxide cements did not significantly affect immediate bond strength, whereas non-eugenol zinc oxide cements adversely affected this property.^40^ Currently, in vivo clinical studies evaluating bond strength and the adhesive interface are not feasible because of methodological limitations. Techniques such as bond strength testing, SEM, and CLSM require tooth extraction for analysis, making these studies incompatible with clinical conditions without compromising patient integrity.

Although residual temporary resin cement remained on the root canal surface and within dentinal tubules, its chemical interaction with the self-adhesive resin cement did not compromise adhesive interface quality. The present study used specimens with circular root canals; however, in oval or flattened canals containing areas inaccessible to burs, mechanical cleaning protocols using UIs and PBs have demonstrated efficacy in removing debris and residual materials. This finding is particularly relevant in inaccessible regions because these protocols enhance resin cement penetration, bond strength, and the clinical reliability of fiberglass post adhesion. Future studies incorporating specimen aging protocols should evaluate the arrangement of dentinal collagen fibers, degradation of temporary and self-adhesive resin cements, and their effects on bond strength.

## Conclusions

Based on the results of this in vitro study, the following conclusions were drawn:

- the temporary resin cement Bifix Temp remained within the dentinal tubules and did not compromise adhesive interface quality, regardless of the mechanical cleaning protocol applied to the root canal;
- Root canal cleaning protocols involving PB or UI significantly enhanced bond strength by nearly 30% and enhanced the intratubular penetration of the self-adhesive resin cement, particularly in the middle and apical thirds, compared with use of the SD alone. These methods proved more effective in removing residual materials and improving cement adaptation to dentin, thereby promoting more reliable adhesion during fiberglass post cementation.

## Data Availability

The datasets generated during and/or analyzed during the current study are available from the corresponding author on reasonable request.
